# Supplementary material for: Novel Driver Strength Index highlights important cancer genes in TCGA PanCanAtlas patients
Source: PeerJ. 2022 Aug 11;10:e13860. doi: 10.7717/peerj.13860 (PMC9375969; doi:10.7717/peerj.13860)
Supplement: Supplemental Information 2 [file peerj-10-13860-s002.zip › COHORTS/genes plots/cohorts/2021_8_16_14_9_distribution_cohorts_LGG.pdf]

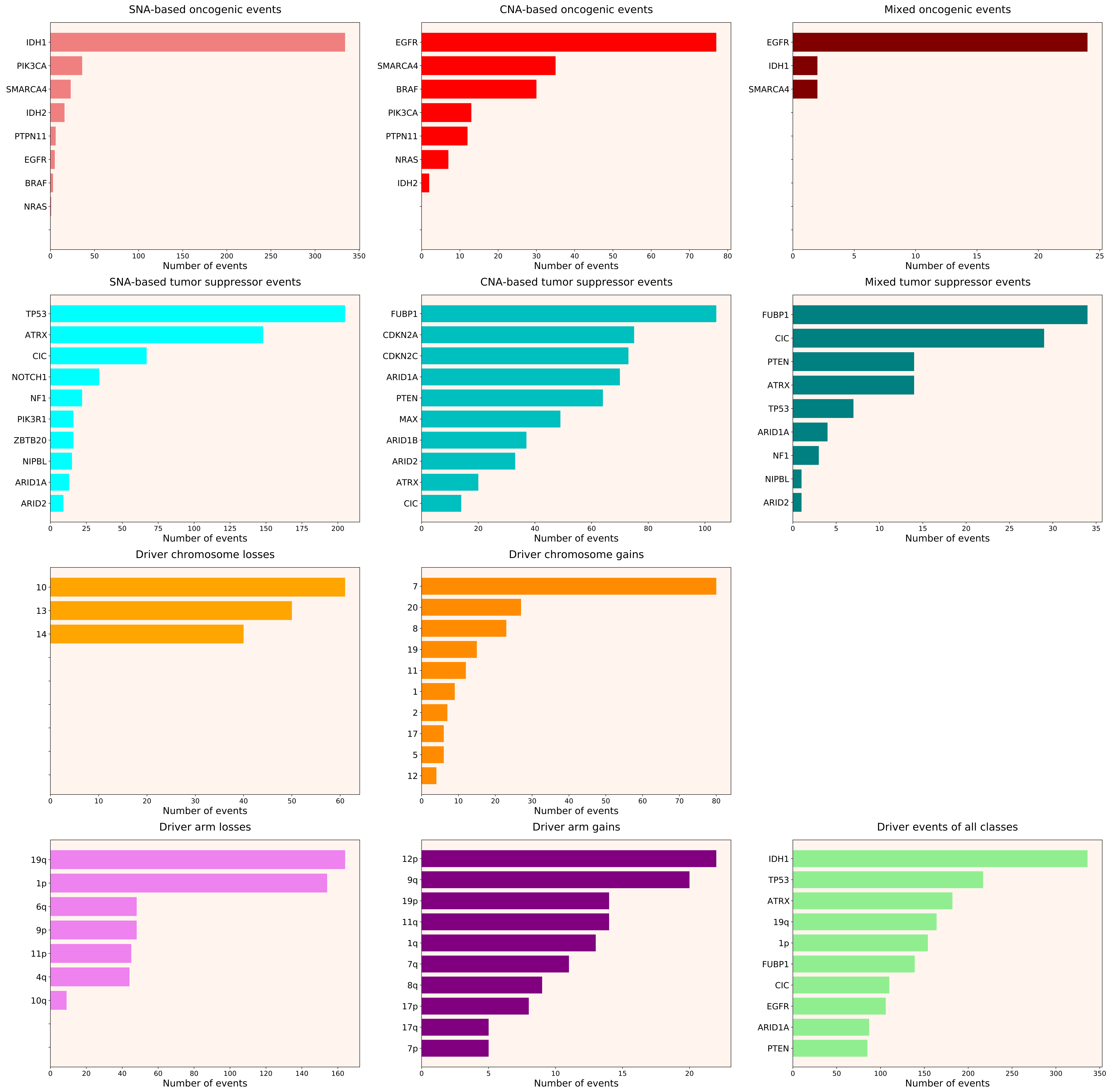

SNA-based oncogenic events
IDH1
PIK3CA
SMARCA4
IDH2
PTPN11
EGFR
BRAF
NRAS
Number of events
CNA-based oncogenic events
EGFR
SMARCA4
BRAF
PIK3CA
PTPN11
NRAS
IDH2
Number of events
Mixed oncogenic events
EGFR
IDH1
SMARCA4
Number of events
SNA-based tumor suppressor events
TP53
ATRX
CIC
NOTCH1
NF1
PIK3R1
ZBTB20
NIPBL
ARID1A
ARID2
Number of events
CNA-based tumor suppressor events
FUBP1
CDKN2A
CDKN2C
ARID1A
PTEN
MAX
ARID1B
ARID2
ATRX
CIC
Number of events
Mixed tumor suppressor events
FUBP1
CIC
PTEN
ATRX
TP53
ARID1A
NF1
NIPBL
ARID2
Number of events
Driver chromosome losses
10
13
14
Number of events
Driver chromosome gains
7
20
8
19
11
1
2
17
5
12
Number of events
Driver arm losses
19q
1p
6q
9p
11p
4q
10q
Number of events
Driver arm gains
12p
9q
19p
11q
1q
7q
8q
17p
17q
7p
Number of events
Driver events of all classes
IDH1
TP53
ATRX
19q
1p
FUBP1
CIC
EGFR
ARID1A
PTEN
Number of events
